# The Accumulation of Phenyllactic Acid Impairs Host Glutamine Metabolism and Inhibits African Swine Fever Virus Replication: A Novel Target for the Development of Anti-ASFV Drugs

**DOI:** 10.3390/v16030449

**Published:** 2024-03-13

**Authors:** Junfei Dai, Xusheng Ma, Ashenafi Kiros Wubshet, Qian Li, Xiaofen Shang, Zhikuan Luo, Jianan Liu, Zhiyu Li, Mingxia Li, Yujie Song, Lijun Guo, Jie Zhang, Haixue Zheng

**Affiliations:** 1 State Key Laboratory for Animal Disease Control and Prevention, College of Veterinary Medicine, Lanzhou University, Lanzhou Veterinary Research Institute, Chinese Academy of Agricultural Sciences, Lanzhou 730000, China; aixinjueluofei@hotmail.com (J.D.); maxusheng@caas.cn (X.M.); nafikw@gmail.com (A.K.W.);; 2 Department of Veterinary Basics and Diagnostic Sciences, College of Veterinary Science, Mekelle University, Mekelle 2084, Ethiopia; 3 China Agricultural Veterinary Bioscience and Technology Co., Ltd., Lanzhou 730046, China

**Keywords:** ASFV, phenyllactic acid, glutamine, metabolics, inhibition

## Abstract

African swine fever (ASF) is a highly contagious and hemorrhagic disease caused by infection with the African swine fever virus (ASFV), resulting in a mortality rate of up to 100%. Currently, there are no effective treatments and commercially available vaccines for ASF. Therefore, it is crucial to identify biochemicals derived from host cells that can impede ASFV replication, with the aim of preventing and controlling ASF. The ASFV is an acellular organism that promotes self-replication by hijacking the metabolic machinery and biochemical resources of host cells. ASFV specifically alters the utilization of glucose and glutamine, which are the primary metabolic sources in mammalian cells. This study aimed to investigate the impact of glucose and glutamine metabolic dynamics on the rate of ASFV replication. Our findings demonstrate that ASFV infection favors using glutamine as a metabolic fuel to facilitate self-replication. ASFV replication can be substantially inhibited by blocking glutamine metabolism. The metabolomics analysis of the host cell after late-stage ASFV infection revealed a significant disruption of normal glutamine metabolic pathways due to the abundant expression of PLA (phenyllactic acid). Pretreatment with PLA also inhibited ASFV proliferation and glutamine consumption following infection. The metabolomic analysis also showed that PLA pretreatment greatly slowed down the metabolism of amino acids and nucleotides that depend on glutamine. The depletion of these building blocks directly hindered the replication of ASFV by decreasing the biosynthetic precursors produced during the replication of ASFV’s progeny virus. These findings provide valuable insight into the possibility of pursuing the development of antiviral drugs against ASFV that selectively target metabolic pathways.

## 1. Introduction

African swine fever (ASF) is a highly contagious and hemorrhagic disease affecting susceptible pigs of all age groups, caused by the African swine fever virus (ASFV). Since its emergence in China in August 2018, ASF has rapidly spread throughout the country, impacting the pig industry [1]. Global efforts have been made to create a safe and effective vaccine against ASF, with a particular focus on understanding the infection and pathogenic mechanism of ASFV [2]. Nevertheless, there have been limited investigations into the correlation between host metabolism and ASFV infection. It has been reported that host metabolic processes play a crucial role in the development of infectious diseases [3,4,5,6,7]. In other words, viruses can exploit cellular metabolites to complete their life cycles, while viral infections can also disrupt metabolic processes in various organs, tissues, and cells. Lastly, metabolites and metabolic reactions can influence viral infection by regulating the host immune response [8,9].

In short, maintaining metabolic homeostasis is the basis for the body to perform a normal physiological function, and an imbalance of metabolic homeostasis can promote a variety of virus infections [10]. During viral replication, there is an increased demand for ATP, biosynthetic precursors, and reducing agents, as viruses lack their own energy and material synthesis systems. By augmenting the energy metabolism and material metabolism of the host organism, ASFV replication is enhanced.

Glucose and glutamine serve as common metabolic fuels for rapidly proliferating cancer cells. Similarly, virus-infected target cells exhibit a predilection for utilizing glucose and glutamine. Glutamine, the predominant amino acid in the bloodstream, undergoes metabolic conversion to α-ketoglutaric acid, which then enters the tricarboxylic acid cycle as part of the feed process. This metabolic pathway is essential for viral mRNA transcription, viral protein synthesis, and viral replication. α-Ketoglutaric acid enters the TCA cycle to generate energy, which can then be converted to pyruvate and lactic acid. It can also reverse the tricarboxylic acid cycle to produce citric acid for lipid synthesis, meeting the needs of cell membranes for rapid division. The TCA cycle not only provides cells with ATP but also precursors for macromolecular synthesis, such as malic acid for gluconeogenesis, NADH for oxidative phosphorylation, and succinyl CoA for ferroheme synthesis. Moreover, glutamine also plays a role in the production of amino acids and nucleotides within cells.

A metabolomics study showed that ASFV infection speeds up viral replication by making the host’s energy and amino acid metabolism work harder in the early stages of infection. Additionally, the buildup of lactic acid inhibits IFNβ and further facilitates viral replication during the later stage of infection [11]. The replication of PRRSV can be controlled by blocking glucose and glutamine metabolism. Epigallocatechin gallate (EGCG) and Telaglenastat (CB-839), which specifically target glutamine metabolism, have been found to effectively suppress the replication of PRRSV [5]. Moreover, supplementing with glutamine can partially rescue viral replication, and using α-ketoglutaric acid (AKG) supplementation, a substance that metabolizes glutamine to compensate for the TCA cycle, can also rescue porcine reproductive and respiratory syndrome virus (PRRSV) replication. These studies collectively indicate that viral infections increase the host’s energy and material metabolism in order to facilitate self-replication. Multiple studies have investigated the significance of glucose and glutamine in functioning as the primary sources of energy for virus-infected cells, which facilitates their self-replication. Moreover, inhibiting the metabolic processes involving glutamine and glucose can effectively impede viral replication [3,4,5,12,13,14].

To identify natural small-molecule compounds that can inhibit ASFV replication by regulating the energy metabolism pathway, we conducted an evaluation of the ASFV infection’s preference for glucose and glutamine metabolism according to the established correlation between the rate of consumption and replication. Using metabolomics, we screened differential metabolites in the later stages of infection, and we confirmed that these metabolites had an effect on the glutamine metabolic pathway and ASFV replication. Our results show a significant correlation between the rate of viral replication and the pace at which glutamine is consumed following ASFV infection. However, the correlation with the rate of glucose consumption was weak. Notably, upon the exhaustion of glucose and glutamine, it was observed that the depletion of glutamine in particular had a notable inhibitory effect on ASFV replication. After being infected with the ASFV, it is recommended to use glutamine as the preferred metabolic substrate. Then, the target cell metabolite phenyllactic acid (PLA) is considerably increased, while glutamine metabolism is suppressed. PLA is an important broad-spectrum antimicrobial compound that inhibits the growth of undesirable microbes through multifaceted action. The mechanisms of PLA-associated microbial inhibition and antivirulence actions have been studied. Similarly, pretreating PLA suppressed the intake of glutamine. Simultaneously, the pathways of nucleotide metabolism and amino acid metabolism that are associated with glutamine were suppressed. Thus, viral replication can be inhibited by reducing the components necessary for the synthesis of progeny viruses.

In conclusion, our findings demonstrate that PLA can impede the glutamine metabolic pathway in the host, which directly hinders ASFV infectivity. This study offers a potential avenue for the development of anti-ASFV drugs.

## 2. Materials and Methods

### 2.1. Cell Culture, Virus, and Reagents

Porcine alveolar macrophages (PAMs) from healthy pigs were harvested and preserved in our laboratory. The PAMs were cultured in an RPMI 1640 medium (Gibco) supplemented with 15% heat-inactivated fetal bovine serum (FBS) and incubated at 37 °C with 5% carbon dioxide (CO_2_). The ASFV CN/GS/2018 strain, belonging to genotype II, was isolated and preserved in the P3 level biosafety laboratory of our institution. The recombinant ASFV with green fluorescence was constructed by the homologous recombination method. In a 6-well culture plate, 5 × 10^6^ cells per well were transfected with 4 ug homologous recombinant plasmid, and ASFV-WT with MOI = 1 was added after 30 min and observed overnight, and the virus was purified using the limited dilution method, named ASFV-GWT. The recombinant strain ASFV-GWT carrying GFP was constructed and kept in the P3 level biosafety laboratory of our institution.

### 2.2. Reagents and Antibodies

Phenyllactic acid (PLA) and 2-deoxy-D-glucose (2DG) were purchased from MedChemExpress (MCE; Monmouth Junction, NJ, USA); a polyclonal antibody against ASFV p72 protein was synthesized in our laboratory as described previously and was used for Western blot assays. Rabbit monoclonal antibody (mAb) against β-actin was purchased from MBL (Nagoya, Japan). Glutamine was purchased from Thermo Fisher Scientific (Waltham, MA, USA). Epigallocatechin gallate (EGCG) was obtained from Aladdin (Shanghai, China). The glucose used in this study was obtained from Sigma-Aldrich (St. Louis, MO, USA).

### 2.3. RNA Extraction and Real-Time Quantitative Reverse-Transcription Polymerase Chain Reaction (qRT-PCR)

Viral RNA was extracted from cultured cells using the TRIzol reagent (Takara, Dalian, China). For the qRT-PCR tests, we used the BioRad CFX96 real-time PCR system in conjunction with a Takara One Step TB Green PrimeScript RT-PCR Kit. The ASFV RNA content was quantified using primers that target the ASFV p72 gene. SLC1A5, GLUT2, GLUT4, and GLS1 RNA content was quantified using the specific primers for each gene. The primer sequences are presented in Table S1.

### 2.4. Western Blot Assay

The samples were vortexed for 10 min at 4 °C after being lysed using RIPA lysis buffer that contained 1 mM of phenylmethylsulfonyl fluoride protease inhibitor (Beyotime, Shanghai, China). The supernatants were then denatured in 5× sample loading buffer at 98 °C for 10 min and subjected to Western blotting. The target proteins were separated by 12% sodium dodecyl sulfate–polyacrylamide gel electrophoresis (SDS-PAGE) and transferred to nitrocellulose membranes (EMD Millipore, Billerica, MA, USA). The membranes were then blocked with 5% skim milk in TBST at room temperature for 2 h and then incubated with an appropriate primary antibody (1:1000) overnight at 4 °C and a secondary antibody (1:5000) at room temperature for 2 h. Antibody–antigen complexes were visualized with chemiluminescence detection reagents (Share-bio Biotechnology, Shanghai, China).

### 2.5. Metabolite Extraction and Data Acquisition through LC-MS Analysis

The metabolic analysis of cells infected with ASFV and control for 24 h was carried out by the APTBIO firm in Shanghai, China, using LC-MS technology. The culture medium from the cultured PAM cells (~10^7^ cells per sample) was removed using a pipette. Then, the cells were washed with PBS under 37 °C, and the PBS was removed. Then, 800 μL of cold methanol/acetonitrile (1:1, *v*/*v*) was added to remove the protein and extract the metabolites. The mixture was collected into a new centrifuge tube, and centrifugation was performed at 14,000× *g* for 5 min at 4 °C to collect the supernatant. The supernatant was dried in a vacuum centrifuge. For LC-MS analysis, the samples were redissolved in 100 μL of acetonitrile/water (1:1, *v*/*v*) solvent. For studying the untargeted metabolomics of polar metabolites, the extracts were analyzed using a quadrupole time-of-flight mass spectrometer (Sciex TripleTOF 6600) with the spectrometry method coupled to hydrophilic interaction chromatography via electrospray ionization at Shanghai Applied Protein Technology Co., Ltd. (Shanghai, China) LC separation was accomplished on an ACQUIY UPLC BEH Amide column (2.1 mm × 100 mm, 1.7 µm particle size (waters, Ireland) using a gradient of solvent A (25 mM ammonium acetate and 25 mM ammonium hydroxide in water) and solvent B (acetonitrile). The gradient was 85% B for 1 min and was linearly reduced to 65% in 11 min; then, it was reduced to 40% in 0.1 min and kept for 4 min and then increased to 85% in 0.1 min, with a 5 min re-equilibration period employed. The flow rate was 0.4 mL/minute, the column temperature was 25 °C, the autosampler temperature was 5 °C, and the injection volume was 2 µL. The mass spectrometer was operated in both negative and positive ion modes. The ESI source conditions were set as follows: ion source gas 1 (Gas1) as 60; ion source gas 2 (Gas2) as 60; curtain gas (CUR) as 30; source temperature, 600 °C; IonSpray Voltage Floating (ISVF), ±5500 V. In MS acquisition, the instrument was set to acquire over the *m*/*z* range of 60–1000 Da, and the accumulation time for the TOF MS scan was set at 0.20 s/spectra. In auto MS/MS acquisition, the instrument was set to acquire over the *m*/*z* range of 25–1000 Da, and the accumulation time for the product ion scan was set at 0.05 s/spectra. The product ion scan was acquired using information-dependent acquisition (IDA) with a high-sensitivity mode selected. The parameters were set as follows: the collision energy (CE) was fixed at 35 V with ±15 eV; the declustering potential (DP) was 60 V (+) and −60 V (−); isotopes within 4 Da were excluded, and the number of candidate ions to monitor per cycle was 10.

### 2.6. Data Analysis

The raw MS data (wiff.scan files) were converted to MzXML files using ProteoWizard MSConvert before importing them into freely available XCMS^plus^ software. For peak picking, the following parameters were used: centWave *m*/*z* = 25 ppm, peak width = c (10, 60), and prefilter = c (10, 100). For peak grouping, bw = 5, mzwid = 0.025, and minfrac = 0.5 were used. In the extracted ion features, only the variables having more than 50% of the nonzero measurement values in at least one group were kept. Compound identification of metabolites by MS/MS spectra with an in-house database established with available authentic standards. After normalization to total peak intensity, the processed data were uploaded before importing into SIMCA-P (version 14.1, Umetrics, Umea, Sweden), where they were subjected to multivariate data analysis, including Pareto-scaled principal component analysis (PCA) and orthogonal partial least-square discriminant analysis (OPLS-DA). Seven-fold cross-validation and response permutation testing were used to evaluate the robustness of the model. The variable importance in the projection (VIP) value of each variable in the OPLS-DA model was calculated to indicate its contribution to the classification. Significance was determined using an unpaired Student’s *t*-test. A VIP value of > 1 and *p* < 0.05 were considered statistically significant.

### 2.7. Bioinformatics Analysis

For KEGG pathway annotation, the metabolites were blasted against the online Kyoto Encyclopedia of Genes and Genomes (KEGG) database to retrieve their COs and were subsequently mapped to pathways in KEGG11. The corresponding KEGG pathways were extracted. To further explore the impact of differentially expressed metabolites, enrichment analysis was performed. KEGG pathway enrichment analyses were performed using Fisher’s exact test, considering the whole metabolites of each pathway as the background dataset. Only pathways with *p*-values under a threshold of 0.05 were considered as significantly changed pathways. For hierarchical clustering, Cluster 3.0 (http://bonsai.hgc.jp/~mdehoon/software/cluster/software.htm) (accessed on 1 November 2022) and the Java Treeview 6.0 software (http://jtreeview.sourceforge.net) (accessed on 1 November 2022) were used. The Euclidean distance algorithm for similarity measurement and the average linkage clustering algorithm (the centroids of the observations were used for clustering) were chosen to perform hierarchical clustering. A heatmap was often generated as a visual aid in addition to dendrograms.

### 2.8. Measurement of Glucose and Glutamine Consumption

The rates of glucose and glutamine consumption were determined using a Glucose Content Assay Kit (Solarbio, Beijing, China) and a Glutamine (Gln) Content Assay (Solarbio, Beijing, China), respectively.

### 2.9. Enzyme Activity Analysis

Glucokinase and glutaminase-1 activity was measured using a Glucokinase Activity Assay Kit (Geruisi-Bio, Suzhou, China) and a Glutaminase (GLS) Activity Assay Kit (Solarbio, Beijing, China).

## 3. Results

### 3.1. Glucose and Glutamine Are Important for ASFV Replication in the Host

**Figure 1:**
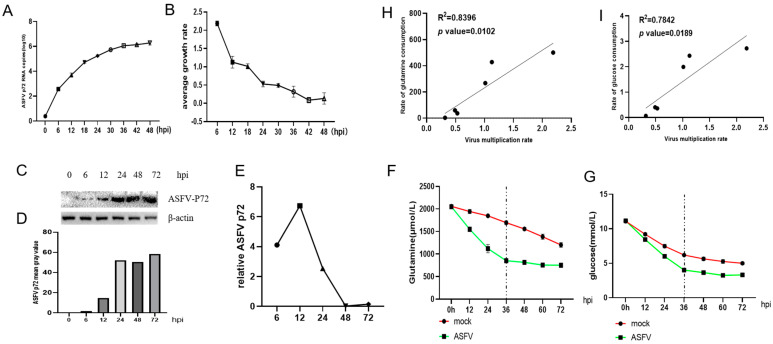
The results demonstrate that glucose and glutamine are important for ASFV replication in PAMs. In a 6-well cell culture plate, 5 × 10^6^ cells were inoculated per well and infected with ASFV with MOI = 1; samples were collected at a specific point in time: (**A**) the qPCR assay was used to measure the RNA copy number of the P72 gene at a certain time point of infection with African swine fever virus (ASFV) to determine its viral replication and proliferation curve; (**C**) the ASFV-P72 protein analysis using WB; (**D**) ImageJ 1.8.0 was used to calculate the average gray values (**B**), and RNA replication and protein expression rates (**E**) of the ASFV-p72 gene were estimated using the RNA copy number in (**A**) and the average gray values in (**D**); (**F**,**G**) the rate at which glutamine and glucose were consumed; the glutamine and glucose levels in the medium supernatant were quantified using commercially available assays. The Pearson linear correlation coefficient (**H**,**I**) was used to illustrate the association between the rates of glucose and glutamine consumption and the rates of ASFV replication and proliferation; The data represent the average ± standard error of the mean (SEM) values obtained from a minimum of three biological replicates.

The qPCR results indicate a progressive increase in the number of p72 protein copies during ASFV infection, as depicted in Figure 1A. Simultaneously, the rate of ASFV replication steadily decreased, as illustrated in Figure 1B. According to Western blotting (WB), although the expression level of the ASFV p72 protein started early in the infection, it increased very slowly and even stopped as the infection progressed (Figure 1C). The WB data were further examined using ImageJ 1.8.0 software; the average gray values depicted in Figure 1D,E lead us to a similar interpretation as that of WB. The correlation analysis revealed a significant association (R^2^ = 0.8396, *p* = 0.0102) between the replication rate of ASFV and the consumption rate of glutamine (Figure 1H), while the replication rate of ASFV was significantly associated with the glucose consumption rate (R^2^ = 0.7842, *p* = 0.0189) (Figure 1I), suggesting that glucose and glutamine uptake by PAM cells is important for ASFV replication in the host.

### 3.2. The Replication of ASFV Is Regulated by the Metabolic Pathways of Glutamine and Glucose

**Figure 2:**
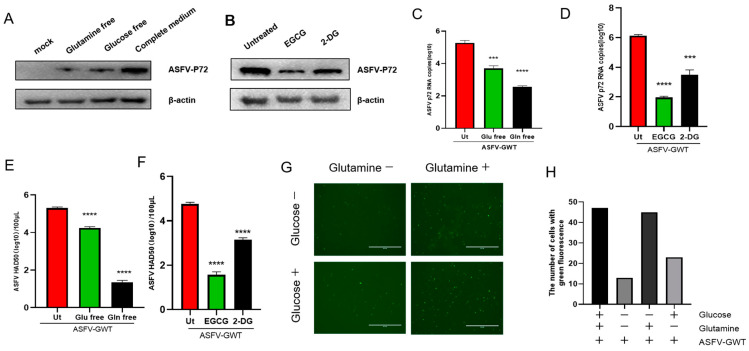
The replication of ASFV is regulated by the metabolic pathways of glutamine and glucose. PAM cells were infected with the African swine fever virus (ASFV) at a multiplicity of infection (MOI) of 1 and subsequently incubated for 24 h with various components. A medium devoid of glutamine, a medium devoid of glucose, and a medium containing all necessary components were used. The protein p72 expression level was determined using Western blot analysis (**A**), the RNA copy number of the ASFV-p72 gene was quantified using quantitative polymerase chain reaction (qPCR) (**C**), and the number of infectious virus particles was determined using the hemadsorption(HAD50) assay (**E**). Fluorescent microscopy (**G**) was used to observe the number of cells infected with ASFV-GWT. The number of cells carrying green fluorescence was calculated using ImageJ 1.8.0 software, and the data were processed using GraphPad Prism 8 software (**H**). PAM cells were infected with the African swine fever virus (ASFV) at a multiplicity of infection (MOI) of 1 in a full culture medium. Subsequently, the cells were treated with the glutamine metabolism inhibitor EGCG (30 μM) and the glucose metabolism inhibitor 2-DG (15 μM) for a duration of 24 h. The protein p72 expression level was determined using Western blotting (**B**), the RNA copy number of the ASFV-p72 gene was quantified using quantitative polymerase chain reaction (qPCR) (**D**), and the number of infectious virus particles was determined using the hemadsorption (HAD50) method (**F**); *** *p* < 0.01 and **** *p* < 0.001. This statistical analysis was conducted using an unpaired *t*-test. The data represent the average values.

To investigate the impact of glutamine and glucose on ASFV replication, PAMs infected with ASFV were cultured in glutamine-free and glucose-free media. The absence of glutamine in the medium was found to significantly impede the reproduction of ASFV, as illustrated in Figure 2A,C,E,G. Additionally, pretreatment with the glutamine metabolism inhibitor EGCG and the glucose metabolism inhibitor 2DG also greatly decreased ASFV replication, as demonstrated in Figure 2B,D,F. Notably, the inhibitory effect of EGCG on ASFV replication was greater than that of 2-DG.

Although both metabolic pathways were found to affect ASFV replication, the regulation effect of glutamine metabolism on ASFV was significantly higher than that of glucose metabolism.

### 3.3. ASFV Significantly Alters Host Glutamine and Glucose Metabolism after Infection

**Figure 3:**
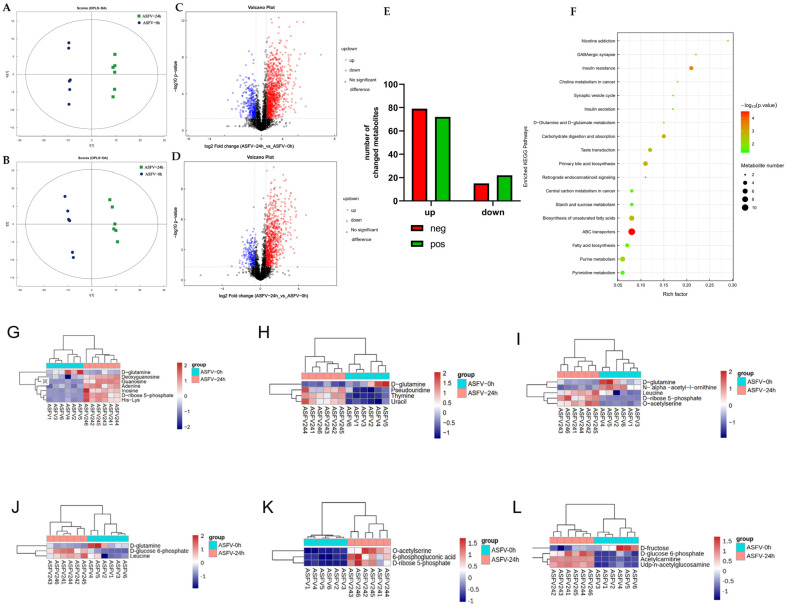
Infection with the African swine fever virus (ASFV) controls the metabolic processes of the host, specifically the metabolism of glutamine and glucose, in order to enhance its own replication. The presence of metabolites resulting from African swine fever virus (ASFV) infection at the 24 h mark, as well as in the control group, was identified using LC-MS. The OPLS-DA model was established from the LC-MS metabolomic profiles of PAM samples (**A**,**B**): (**A**) POS; (**B**) NEG. Volcano plots depict the data for cells infected with ASFV at specific time intervals. Every spot on the volcanic map corresponds to a metabolite. The red color indicates an increase in concentration, blue indicates a decrease in concentration, and gray indicates no change (**C**,**D**): (**C**) POS; (**D**) NEG. The number of metabolites that underwent a considerable increase in concentration (shown in red) and decrease in concentration (shown in blue) in cells infected with ASFV are presented (**E**). The metabolites exhibiting significant changes were analyzed using a histogram (Figure S1A,B): (S1A) POS; (S1B) NEG. The red color indicates an increase in activity, whereas green indicates a decrease in activity. The *X*-axis indicates the factor of variation. Every column corresponds to a metabolite. Bubble plots for metabolic pathway analysis of cells infected with the African swine fever virus (ASFV) are shown (**F**). Each bubble in the bubble diagram represents a metabolic pathway. The top 20 pathways with the highest significance were chosen based on the *p*-value. The size of the bubble is directly proportional to the number of metabolites. The *X*-axis represents the pathway effect value in the topological study, and its magnitude is positively correlated with the influence factor. The *Y*-axis represents the *p*-value of the metabolic pathway in the enrichment study. A darker color indicates a smaller *p*-value, which signifies a more significant enrichment degree. The heatmap displays the results of hierarchical clustering analysis for the differential metabolites associated with the glutamine metabolic route (**G**–**I**) and the glucose metabolic pathway (**J**–**L**). Each column corresponds to a single sample, and each row corresponds to a distinct differential metabolite. The red color represents an increase in concentration, while blue represents a decrease in concentration.

As the infection progressed to its late stages, glutamine consumption and ASFV replication proportionally ceased. An OPLS-DA (orthogonal partial least-square discriminate analysis) model was used to assess the liquid chromatography–mass spectrometry (LC/MS) metabolomic profiles of PAM samples in order to identify the chemicals that hinder glutamine metabolism during infection (Figure 3A,B).

Briefly, 24 h after infection, both ASFV-infected cells and mock were subjected to LC-MS metabolomic profiling. According to the LC/MS metabolomic analysis, the differential metabolites were visualized using volcano plots. Each point on the plot represents a metabolite. Fold changes greater than 1.5 or less than 0.67 and a *p*-value of less than 0.05 from the Student’s *t*-test are displayed. The points presented with red and blue color in the volcano plot show metabolites that increased and decreased in concentration, respectively. On the other hand, there was a significant variation (*p* < 0.05) in the metabolites’ cationic and anion sources (Figure S1A,B). Accordingly, each column represents a metabolite; red indicates an increase in concentration, green indicates a decrease in concentration, and the horizontal coordinate denotes the difference multiple.

Among the metabolites identified using an anion source, 79 were considerably increased, and 15 were significantly decreased in concentration (Figure 3E). PLA was the most increased metabolite, as shown in Figure S1B. Similarly, cationic sources revealed 72 metabolites that increased in concentration and 22 that decreased in concentration (Figure 3E). Lower levels of the metabolite glutamine were observed later in the metabolic pathway, as shown in Figure S1A. Additionally, Figure 3F displays the results of the KEGG pathway enrichment analysis of the differential metabolites observed during the late stage of ASFV infection. In this analysis, the size of each bubble corresponds to the number of enriched metabolites in the pathway, while the color of the bubble indicates the magnitude of the *p*-value. The top 20 pathways with the highest significance were chosen based on the *p*-value. The figure displays the several metabolites involved in the glutamine metabolic route, including purine metabolism, amino acid biosynthesis, and pyrimidine metabolism (Figure 3G–I). It also illustrates the several metabolites involved in the glucose metabolic route, including carbon metabolism, insulin resistance, and central carbon metabolism in cancer (Figure 3J–L).

In the glutamine metabolic pathway, the levels of other associated metabolites were predominantly increased in concentration. According to the metabolic bottleneck analysis, the overall maximum metabolic flux of a complete metabolic pathway was limited by metabolites with the smallest metabolic flux, suggesting that the inhibition of glutamine leads to an overall decrease in the related metabolic pathways.

### 3.4. Pretreatment with PLA Results in a Decrease in Glutamine and Glucose Intake and a Reduction in ASFV Replication

**Figure 4:**
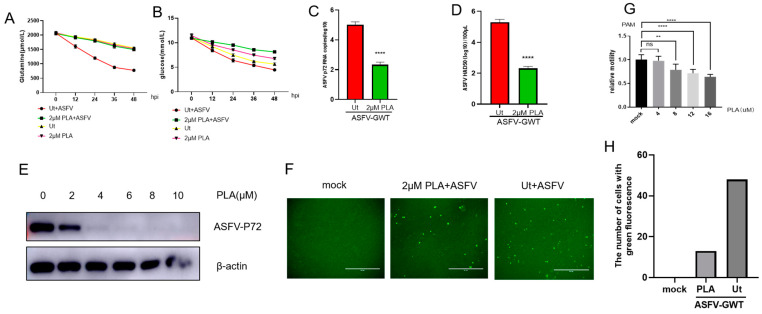
Pretreatment with PLA results in a decrease in glutamine and glucose intake and a reduction in ASFV replication. PAM cells were exposed to a predetermined amount of PLA for a duration of 24 h. The impact of PLA on the viability of PAM cells was assessed using a commercially available CCK8 cell activity detection kit, (ns) *p* value > 0.05, (**) *p* value < 0.01, (****) *p* value < 0.0001 (**G**). The rates of glutamine and glucose consumption and the glutamine and glucose levels in the medium supernatant were quantified using commercially available kits (**A**,**B**). The suppression of ASFV replication by PLA was assessed using Western blot analysis to detect the expression of p72 protein (**E**), quantitative polymerase chain reaction (qPCR) was performed to measure the copy number of ASFV-P72 gene RNA (**C**), and hemadsorption (HAD50) assay was used to determine the number of infectious virus particles (**D**). The fluorescent microscopy technique was used to observe the number of cells infected with ASFV-GWT (**F**). The number of cells carrying green fluorescence was calculated using ImageJ 1.8.0 software, and the data were processed using GraphPad Prism 8 software (**H**).

According to the metabolite analysis, in the late stage of ASFV infection, the accumulation of a large amount of PLA significantly reduces glutamine metabolism. An experiment was conducted to examine the interaction between PLA and glutamine metabolism. This was achieved by qualitatively detecting the consumption of glutamine in PAM cells that were pretreated with PLA and infected with ASFV. The cytotoxicity of phenyllactic acid (PLA) was assessed using the CCK8 assay. PLA with a dose of less than 4 μM exhibited no significant cytotoxicity to PAM cells, as shown in Figure 4G. Glutamine and glucose uptake by target cells were measured. PLA pretreatment significantly reduced the consumption of glutamine (Figure 4A) and glucose (Figure 4B). Moreover, PLA pretreatment significantly downregulated the transcription (Figure 4C) and protein expression (Figure 4E) levels of the ASFV-P72 gene. HAD50 detection showed that PLA could reduce the number of progeny virions (Figure 4D). The results of fluorescence microscopy revealed the number of virus-infected cells with GFP expression (Figure 4F). Therefore, pretreatment with PLA can decrease glutamine consumption and hinder viral multiplication. Further research is required to evaluate the impact of PLA on the glutamine metabolic pathway.

### 3.5. ASFV Replication Is Hindered by PLA through a Reduction in Metabolic Flux in Glutamine and Glucose Metabolism Pathways

**Figure 5:**
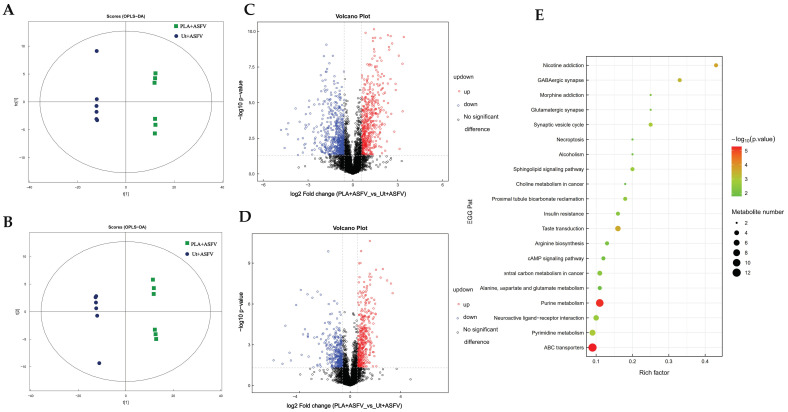

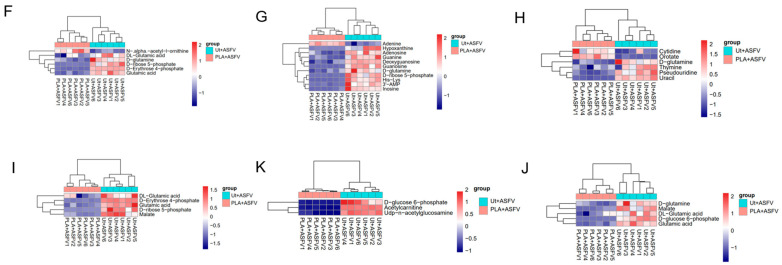
ASFV replication is hindered by PLA through a reduction in metabolic flux in glutamine and glucose metabolic pathways. The LC-MS technique was used to detect the metabolites in PAM cells infected with ASFV for 24 h. The cells were divided into two groups: One group was pretreated with 4Μm, and the other group was untreated. The OPLS-DA model was established using the LC-MS metabolomic profiles of PAM samples (**A**,**B**): (**A**) POS; (**B**) NEG. Volcano plots depicting the data for cells infected with ASFV at specified time intervals are presented. Every spot on the volcanic map corresponds to a metabolite. The red color indicates an increase in concentration, blue indicates a decrease in concentration, and gray indicates no change (**C**,**D**): (**C**) POS; (**D**) NEG. The metabolites exhibiting significant changes were analyzed using a histogram (Figure S2A,B). The analysis of metabolites was performed separately for cationic source detection (POS) (Figure S2A) and anion source detection (NEG) (Figure S2B). The red color indicates an increase in activity, whereas green indicates a decrease in activity. The *X*-axis indicates the factor of variation. Every column corresponds to a metabolite. The bubble plots for metabolic pathway analysis of cells infected with the African swine fever virus (ASFV) are shown (**E**). Each bubble in the bubble diagram corresponds to a metabolic pathway. The top 20 pathways with the highest significance were chosen based on the *p*-value. The size of the bubble is directly proportional to the amount of metabolites. The *X*-axis represents the value of pathway impact in the topological analysis, and its magnitude is directly proportional to the influence factor. The *Y*-axis represents the *p*-value of the metabolic route in the enrichment study. A deeper color indicates a smaller *p*-value and thus a higher degree of significance for the enrichment. A heatmap was generated using the hierarchical clustering technique to visualize the correlation between differential metabolites and the glutamine metabolic pathway (**F**–**H**), as well as the correlation with the glucose metabolic pathway (**I**–**K**). Each column corresponds to a single sample, and each row corresponds to a distinct differential metabolite. The red color represents an increase in concentration, while blue represents a decrease in concentration.

The OPLS-DA model showed that PLA pretreatment could significantly change the metabolism of the treated group and the control group (Figure 5A,B). The volcanic plots display all the distinct metabolites (Figure 5C,D), whereas Figure S2A,B illustrate the important variations in metabolites. The KEGG analysis revealed that, following PLA pretreatment, differential metabolites were primarily associated with metabolic pathways related to glutamine and glucose (Figure 5E). The analysis of differential metabolites in interconnected metabolic pathways revealed that PLA pretreatment had a significant impact on reducing the levels of metabolites in those pathways. The metabolomics investigation of PAM cells infected with the ASFV and pretreated with PLA revealed a significant reduction in glutamine metabolism (Figure 5F–H) and the inhibition of glucose metabolic pathways (Figure 5I–K) in the host cells. These related metabolic pathways are capable of synthesizing biosynthetic precursors, including nucleotides and amino acids, that are used for ASFV replication.

### 3.6. PLA Affects the Activities of Transporters and Related Enzymes in Glutamine and Glucose Metabolic Pathways

**Figure 6:**
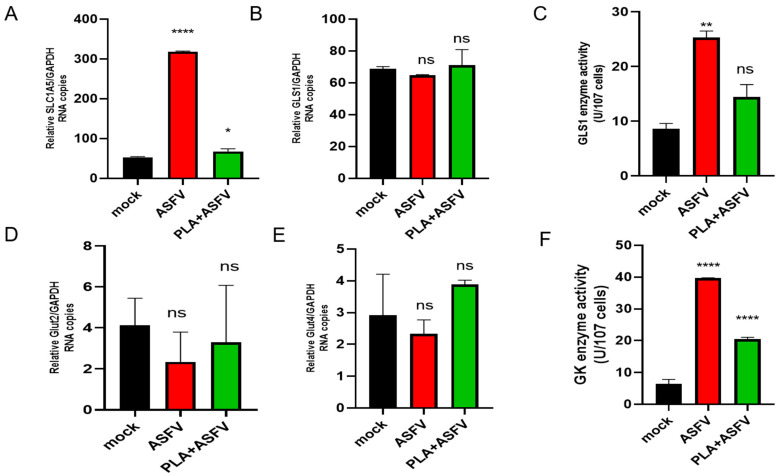
PLA affects the transportation of glutamine and glucose, as well as the activity of enzymes associated with them. PAM cells, which were pretreated with a concentration of 2 μM PLA, were infected with the African swine fever virus (ASFV) at a multiplicity of infection (MOI) of 1. After a 24 h period, samples were obtained, and total RNA was extracted. The RNA transcription levels of glutamine transporter SLC1A5 (**A**), glutaminase GLS1 (**B**), glucose transporter Glut2 (**D**), and glucose transporter Glut4 (**E**) were determined by qPCR. Enzyme activity (**C**) of GLS1 and enzyme activity (**F**) of GK were measured using commercial glutaminase GLS1 and glucokinase GK activity detection kits, following the provided instructions; ns *p* > 0.05, * *p* < 0.05, ** *p* < 0.01 and **** *p* < 0.0001; unpaired *t*-test. Data are the mean ± SEM values of at least three biological replicates.

In order to explore the mechanism of PLA inhibiting glutamine and glucose metabolism, we determined the transcriptional level of glutamine transporter SLC1A5 (Figure 6A) and GLS (Figure 6B), and the enzyme activity of GLS (Figure 6C), a key enzyme in glutamine metabolism. The results showed that PLA could significantly reduce the transcription level of SLC1A5 and the enzyme activity of GLS but did not significantly change the transcription level of GLS. The analysis of the transcription level of the transporters GLUT2 (Figure 6E) and GLUT4 (Figure 6F), as well as the activity of GK (Figure 6F), a crucial enzyme in the glucose metabolic pathway, revealed that PLA did not have a significant impact on the transcription level of these transporters. However, it was found that PLA could decrease the enzyme activity of GK.

## 4. Discussion

Blocking glutamine metabolism can significantly inhibit ASFV replication. After infection, host glutamine metabolism is weakened, and viral replication is inhibited. The metabolomics analysis of differential metabolites showed that ASFV infection induced metabolic reprogramming in the host, and the metabolite PLA accumulated in large quantities in the late stage of infection, while glutamine metabolism and the related metabolic pathways were weakened. Phenyllactic acid (PLA) is an important broad-spectrum antimicrobial compound that inhibits the growth of undesirable microbes through multifaceted actions. The mechanisms of PLA-associated microbial inhibition and antivirulence actions have been studied. PLA pretreatment facilitated the inhibition of ASFV replication and glutamine consumption by PLA. Further metabolomics analysis was performed to investigate the effect of PLA on ASFV hijacking glutamine metabolism to promote self-replication. The results of pretreatment with PLA showed that PLA could significantly inhibit the increased concentration of glutamine induced by ASFV infection and the metabolic flux of related pathways involved in glutamine metabolism. PLA had an influence on key glutamine metabolic pathways by inhibiting the activity of GLS, a key enzyme of the glutamine metabolic pathway, but had no effect on the glucose transporter.

Glucose and glutamine are commonly used metabolic fuels in mammalian cells [15], and the ASFV uses ATP and biosynthetic precursors generated by glucose and glutamine metabolism to promote self-replication after infection [16]. Although an increase in ASFV infection increased the consumption of glutamine and glucose at the same time, the correlation between the metabolic rates of glutamine and glucose and ASFV replication suggests that the catabolism of glutamine is mainly used for self-replication after ASFV infection, which can also be explained by the fact that the effect of glutamine supplementation on saving ASFV replication was significantly higher than that of glucose supplementation. The metabolomics analysis of late-stage ASFV infection showed that a large amount of PLA accumulated in the late stage of ASFV infection, while glutamine metabolism and glutamine-related metabolic pathways were inhibited. It is speculated that the accumulation of PLA leads to the inhibition of glutamine metabolism through the feedback of host metabolic reprogramming. PLA acts as a spectral antifungal and antibacterial metabolite [17,18]. To verify the effect of PLA on viral replication, we used PLA to treat PAM cells, which slowed down the rate of glutamine uptake and stopped the replication of ASFV. This suggests that PLA can be used as a natural small-molecule compound for antiviral purposes. ASFV needs to hijack the host metabolism for its own replication. Nucleotides and amino acids are used as raw materials for the replication and synthesis of progeny viruses, which are related to glutamine metabolism. Therefore, in order to explore the effects, we performed metabolomics analysis, and the results showed that PLA pretreatment significantly reduced the metabolic flux of glutamine, nucleotide metabolism (including purine and pyrimidine metabolism), and amino acid biosynthetic flux, and therefore reduced the raw material for ASFV replication at the feedstock level. Thus, the replication level of ASFV was reduced.

Consistent with previous studies [11], ASFV increased host energy metabolism and amino acid metabolism in the early stage of infection, promoted self-replication, and inhibited amino acid metabolism in the later stage of infection. Using metabolomics, this study revealed that a considerable amount of PLA accumulated at the end of the infection and suppressed glutamine metabolism. This inhibited the amino acid biosynthetic flux and nucleotide biosynthetic flux involved in glutamine metabolism, which indirectly affected ASFV replication.

**Figure 7:**
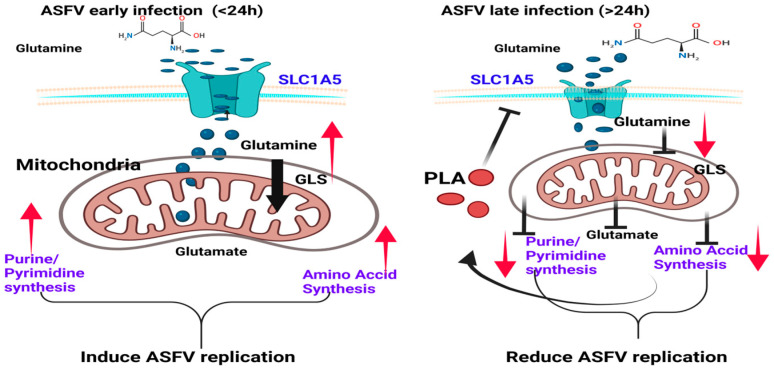
This schematic illustrates how PLA buildup hinders the replication of ASFV by blocking glutamine and its associated metabolic pathways. During the initial phase of infection, ASFV stimulates an increase in glutamine and glucose metabolism in the host, leading to an enhanced flow of metabolic activity in the purine metabolism, pyrimidine metabolism, and amino acid biosynthesis pathways. During the period following infection that occurs after 24 h, the buildup of PLA metabolite hinders ASFV replication by diminishing the function of glutamine transporter and GLS1, the pivotal enzyme in the glutamine metabolic pathway. This, in turn, reduces the metabolic flow of associated purine metabolism, pyrimidine metabolism, and amino acid biosynthesis pathways, as well as the bioprecursor substances utilized for the production of ASFV progeny viruses.

In the early stage of ASFV infection, the virus hijacks and increases the host’s glutamine and glucose metabolism to promote viral replication, while PLA accumulates over time and gradually reduces the transcription of glutamine transporter and the activity of GLS, a key enzyme in glutamine metabolism, in the late stage of ASFV infection, thereby reducing the level of glutamine metabolism. Affected by the decrease in glutamine metabolism, glutamine-related metabolic pathways, including amino acid bio-anabolism and nucleotide metabolism, also weaken. Thus, the biosynthetic precursors used for ASFV replication are reduced to effectively inhibit ASFV replication (Figure 7).

Although PLA can inhibit viral replication by inhibiting glutamine metabolism, it can also be used as a potential antiviral drug. However, for the body, immune cells that require a large number of substances and considerable energy for metabolic functions, including the activation of macrophages and the proliferation and activation of T/B lymphocytes caused by infection, also require a large intake of glucose and glutamine to meet their metabolic needs in terms of energy consumption and substance intake [19,20,21]. We have formulated the following questions to provide valuable insights for future scholars in this field: Does PLA have an inhibitory effect on the activation and proliferation of these immune cells? Can a reduction in the degree of activation and proliferation of immune cells lead to immunosuppression and thus aggravate the disease [22]? More research is needed to determine these issues.

## 5. Conclusions

To summarize, our findings indicate that, upon infection, ASFV hijacks the host’s metabolic functions in order to utilize glutamine as a metabolic substrate for its own replication and proliferation. PLA, a naturally occurring metabolite, can hinder ASFV proliferation by blocking glutamine metabolism. Developing antiviral drugs that target metabolic pathways is a feasible plan. More research into the unique metabolic pathways used by ASFV-infected hosts and other rapidly proliferating immune cells is required to improve the targeting of ASFV-infected cells while simultaneously decreasing the likelihood of adverse reactions.

## Data Availability

Data are contained within the article and Supplementary Materials.

## Supplementary Materials

The following supporting information can be downloaded at: https://www.mdpi.com/article/10.3390/v16030449/s1, Figure S1: Differential metabolites of ASFV infected PAM cells for 24 h. The metabolites exhibiting significant changes were analyzed using a histogram (Figure S1A,B). (S1A) POS and (S1B) NEG. The colour red indicates an increase in activity, whereas the colour green indicates a decrease in activity. The *X*-axis displays the fold change. Every column corresponds to a metabolite; Figure S2: Differential metabolites of PLA pretreated PAM cells infected with ASFV for 24 h. The metabolites exhibiting significant changes were analyzed using a histogram (Figure S2A,B). (S2A) POS and (S2B) NEG. The colour red indicates an increase in activity, whereas the colour green indicates a decrease in activity. The *X*-axis displays the fold change. Every column corresponds to a metabolite; Table S1: Primers of target genes for qPCR detection.
