# Supplementary material for: The Accumulation of Phenyllactic Acid Impairs Host Glutamine Metabolism and Inhibits African Swine Fever Virus Replication: A Novel Target for the Development of Anti-ASFV Drugs
Source: Viruses. 2024 Mar 13;16(3):449. doi: 10.3390/v16030449 (PMC10975624; doi:10.3390/v16030449)
Supplement: Supplementary file 1 [file viruses-16-00449-s001.zip › viruses-2878493-supplementary.pdf]

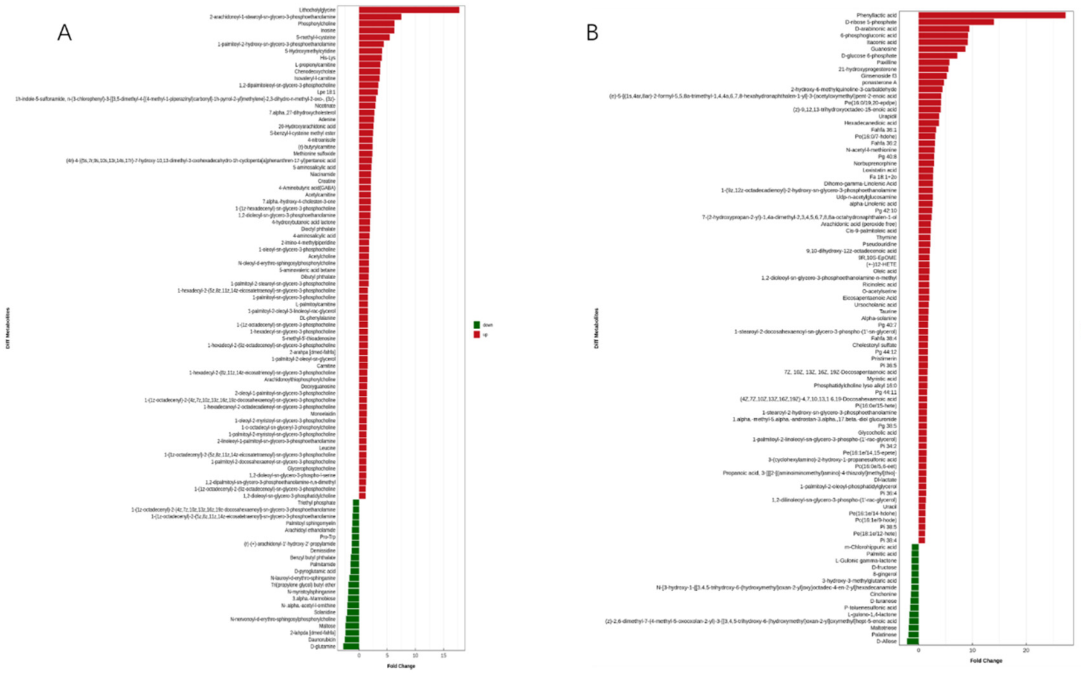

Figure S1. Differential metabolites of ASFV infected PAM cells for 24h. The metabolites exhibiting significant changes were analyzed using a histogram (FigS1A and B). (S1A) POS and (S1B) NEG. The colour red indicates an increase in activity, whereas the colour green indicates a decrease in activity. The X-axis displays the fold change. Every column corresponds to a metabolite.

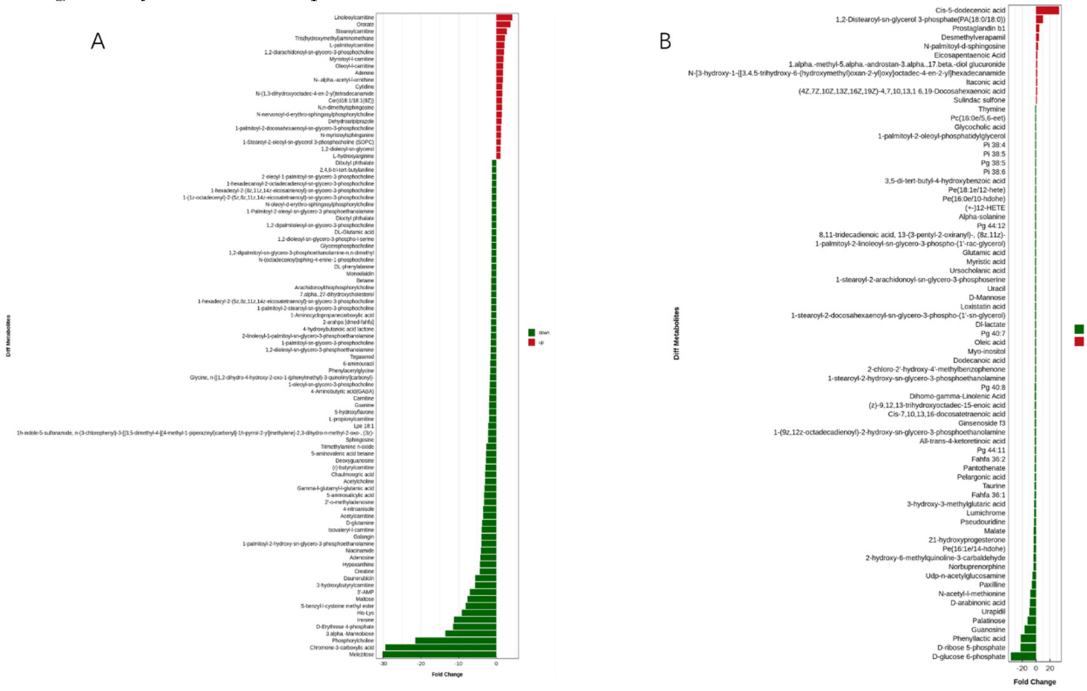

Figure S2. Differential metabolites of PLA pretreated PAM cells infected with ASFV for 24h. The metabolites exhibiting significant changes were analyzed using a histogram (FigS2A and B). (S2A) POS and (S2B) NEG. The colour red indicates an increase in activity, whereas the colour green indicates a decrease in activity. The X-axis displays the fold change. Every column corresponds to a metabolite.

Target gene	Primer sequence
ASFV-P72-F	5- CTGCTCATGGTATCAATCTTATCGA-3
ASFV-P72-R	5- GATACCACAAGATCAGCCGT-3
SLC1A5-F	5-CTGTTCCCTCCCGGGATTCT-3
SLC1A5-R	5-TCCGAAAAGCTCCAGACGAC-3
GLS1-F	5-TGTCATGGGCATGATGTGCT-3
GLS1-R	5-AAGGAATGCCTTTGATCACCA-3
GLUT2-F	5-CAGACAGCTGGAATCAGCCA-3
GLUT2-R	5-CACAAGTCCCACCGACATGA-3
GLUT4-F	5-GGCCATCGTCATTGGCATTC-3
GLUT4-R	5-GTCAGGCGCTTCAGACTCTT-3
GAPDH-F	5-TCGGAGTGAACGGATTTGGC-3
GAPDH-R	5-TGACAAGCTTCCCGTTCTCC-3

Table S1. Primers of target genes for qPCR detection.
